# Heart Rate Variability-Guided Training for Improving Mortality Predictors in Patients with Coronary Artery Disease

**DOI:** 10.3390/ijerph191710463

**Published:** 2022-08-23

**Authors:** Agustín Manresa-Rocamora, José Manuel Sarabia, Silvia Guillen-Garcia, Patricio Pérez-Berbel, Beatriz Miralles-Vicedo, Enrique Roche, Néstor Vicente-Salar, Manuel Moya-Ramón

**Affiliations:** 1 Department of Sport Sciences, Sports Research Centre, Miguel Hernández University of Elche, 03202 Elche, Spain; 2 Institute for Health and Biomedical Research of Alicante (ISABIAL), 03010 Alicante, Spain; 3 Department of Cardiology, Hospital General de Elda, 03600 Elda, Spain; 4 Department of Cardiology, Hospital Clínico Universitario San Juan, 03550 San Juan de Alicante, Spain; 5 Department of Cardiology, Hospital Universitario del Vinalopó, 03293 Elche, Spain; 6 Department of Applied Biology-Nutrition, Institute of Bioengineering, Miguel Hernández University of Elche, 03202 Elche, Spain; 7 CIBER Fisiopatología de la Obesidad y Nutrición (CIBEROBN), Instituto de Salud Carlos III (ISCIII), 28029 Madrid, Spain

**Keywords:** cardiac rehabilitation, cardiorespiratory fitness, heart rate-based indices, methodological issues

## Abstract

The objective of this research was to investigate whether heart rate variability (HRV)-guided training improves mortality predictors to a greater extent than predefined training in coronary artery disease patients. Twenty-one patients were randomly allocated to the HRV-guided training group (HRV-G) or the predefined training group (PRED-G). They measured their HRV at home daily and trained three times a week for six weeks. Resting heart rate, isolated vagal-related HRV indices (i.e., RMSSD, HF, and SD_1_), weekly averaged RMSSD, heart rate recovery, and maximum oxygen uptake were assessed before and after the training period. There was a statistically significant difference (*p* = 0.034) in the change in weekly averaged RMSSD in favor of the HRV-G, while no differences were found in the remaining analyzed variables (*p* > 0.050). Regardless of the training prescription method, exercise training decreased resting heart rate (*p* = 0.001; −4.10 [95% CI = −6.37–−1.82] beats per minute (bpm)), and increased heart rate recovery at 2 min (*p* = 0.010; 4.33 [95% CI = 1.15–7.52] bpm) and maximum oxygen uptake (*p* < 0.001; 3.04 [95% CI = 1.70–4.37] mL·kg^−1^·min^−1^). HRV-guided training is superior to predefined training in improving vagal-related HRV when methodological factors are accounted for

## 1. Introduction

Coronary artery disease (CAD) is one of the deadliest diseases worldwide. Thus, the development of cost-efficient treatments to reduce mortality in CAD patients is mandatory. Accumulating evidence has demonstrated that exercise-based cardiac rehabilitation (CR) programs reduce mortality rates in patients with CAD [1]. In this regard, parasympathetic nervous system (PNS) activity and cardiorespiratory fitness (CRF), assessed by heart rate (HR)-based indices (e.g., resting HR, resting HR variability (HRV), and post-exercise HR recovery (HRR)) and maximum oxygen uptake (VO_2_ max), respectively, are considered independent predictors of mortality [2,3,4,5,6]. Moreover, Martinez et al. [7] and Medeiros et al. [8] reported an improvement in PNS activity after an exercise-based CR program. In a recent systematic review and meta-analysis, Manresa-Rocamora et al. [9] demonstrated that exercise-based CR programs also enhance VO_2_ max. Thus, the reduction in mortality rates could be explained in part by the training-induced effect on mortality predictors (i.e., HR-based indices and VO_2_ max).

Within HR-based indices, resting HR, resting HRV, and HRR are commonly used as non-invasive reliable tools to indirectly measure the PNS status [10,11]. HRV is defined as the oscillation in the interval between heartbeats, and it depends on the continuous modulation of the autonomous nervous system (ANS) branches [12]. Higher HRV represents higher levels of PNS modulation, whereas lower HRV signifies greater modulation of the sympathetic nervous system (SNS) and is associated with a higher risk of mortality [5,13]. Within HRV indices, the root-mean-square difference of successive normal R-R intervals (RMSSD), the high frequency (HF; 0.15–0.40 Hz), and the standard deviation of instantaneous beat-to-beat R-R interval variability (SD_1_) are considered vagal-related HRV indices, while the interpretation of other HRV indices is more difficult [10,14,15,16]. On the other hand, post-exercise HRR exhibits a first-order exponential decay function with a rapid fall in HR immediately after exercise (i.e., 1 min) occurring primarily due to PNS reactivation. In contrast, there is evidence that long-term HRR (i.e., 2–5 min) is associated with PNS reactivation and SNS withdrawal [17]. Therefore, an increase in HRR during the first minute after exercise represents an increase in PNS reactivation, whereas interpretation of long-term HRR is more difficult. Although there is evidence that exercise-based CR improves vagal-related HRV and HRR indices in patients with CAD [18], there are contradictory findings on the training-induced effect on PNS modulation based on the vagal-related HRV index used [18]. In this regard, previous studies revealed the influence of methodological issues on the sensitivity of vagal-related HRV indices for inferring PNS modulation [19]. For instance, there is evidence that day-to-day RMSSD values averaged across several days (i.e., averaged RMSSD) are more suitable than other isolated vagal-related HRV indices to reflect PNS activity due to the fact that averaged values reduce the natural day-to-day lability of HRV indices [20].

On the other hand, even though important benefits have been reported after an exercise-based CR program, the inter-individual responsiveness varies considerably among patients if a predefined training program is applied. In the latter, training variables (e.g., training intensity) are set prior to the start of the intervention [21]. This variability in the training responses could be due to factors such as age, gender, initial CRF, or race [9,22]. Moreover, the PNS function could also play a determinant role in the responsiveness to exercise training. For instance, Hedelin et al. [23] and Compostella et al. [24] found lower values of vagal-related HRV indices at baseline associated with lower CRF improvement in sedentary subjects and chronic heart failure (CHF) patients, respectively. Moreover, HRV is considered a valid marker to reflect training-induced status [25]. For these reasons, HRV has also been used to carry out aerobic training prescriptions, known as HRV-guided training, in endurance-trained athletes [26,27] and sedentary people [28].

The basic idea behind HRV-guided training is to adjust the training stimulus when PNS activity differs meaningfully from the reference values (i.e., higher than the smallest worthwhile change (SMC)) [29]. Previous studies reported that HRV-guided training is superior to predefined training in improving PNS activity and CRF [28,30,31]. Moreover, the individual response to HRV-guided training seems less heterogeneous [30,32]. In this regard, the individual response to training is also heterogeneous in CAD patients [21]. Moreover, CAD patients are often prescribed beta-blockers to treat autonomic dysfunction (e.g., diminished PNS activity), and these drugs are associated with altered response to HRV-guided training. Behrens, Hottenrott, Weippert, Montanus, Kreuzfeld, Rieger, Lübke, Werdan and Stoll [31] found that HRV-guided training is better than predefined training for improving CRF in CHF patients. Conversely, the effect of exercise training, based on the training prescription method, on PNS modulation is yet to be explored. Therefore, the main objective of this research was to determine whether HRV-guided training increases HR-based indices or VO_2_ max to a greater extent than predefined training in CAD patients. Secondary objectives included testing the effect of HRV-guided training versus predefined training on secondary outcomes (e.g., body composition, quality of life), and studying the effect of exercise-based CR, regardless of the training prescription method, on mortality predictors and secondary endpoints. Based on previous evidence, it was hypothesized that HRV-guided training is superior to predefined training in improving mortality predictors in CAD patients. Moreover, regardless of the training prescription method, we hypothesized that exercise-based CR should improve mortality predictors in CAD patients.

## 2. Materials and Methods

### 2.1. Study Design, Randomization, and Implementation

**Figure 1:**
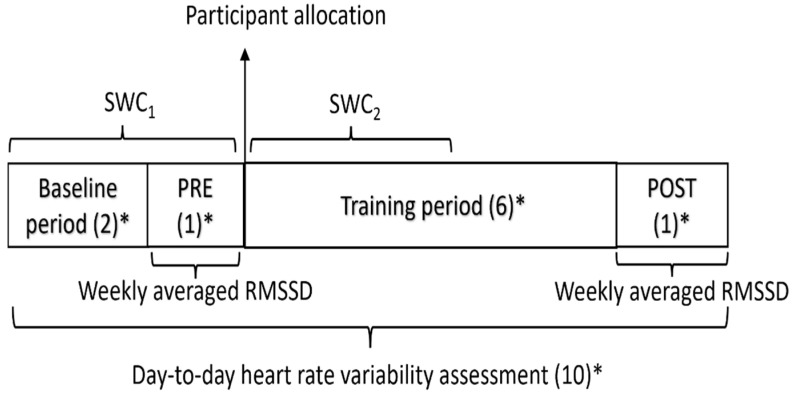
Experimental design. PRE, baseline assessment week; POST, final assessment week; RMSSD, the root-mean-square difference of successive normal R-R intervals; SWC_1_, smallest worthwhile change during the first three weeks of the study protocol; SWC_2_, smallest worthwhile change during the first three weeks of the training period; * denotes number of weeks.

This study was a parallel-group, double-blind, randomized controlled trial that was performed from October 2018 to July 2019. Before taking part in the study, patients were interviewed and signed written informed consent. In brief, patients were given instructions on how to carry out day-to-day HRV measurements. The study protocol was divided into two periods: a two-week baseline period (BP) and a six-week training period (TP). A baseline assessment week (PRE) and a final assessment week (POST) were conducted before and after the TP, respectively. The patients were matched into pairs according to their VO_2_ max, waiting time to start CR, gender, and peak power output at PRE. Afterwards, the patients were randomly allocated to an HRV-guided training group (HRV-G) or predefined training group (PRED-G) (Figure 1). An allocation sequence was computer-generated following a non-blocked strategy and delivered by a researcher not involved in the trial. A researcher who was aware of the study design conducted the enrolment and assignment of the patients. Throughout the TP, patients allocated to the PRED-G carried out a predefined training program, while patients that were assigned to the HRV-G trained based on their day-to-day HRV measurements. Several assessments took place at PRE and POST for each patient, all performed in the same order and at the same time of day. Patients and assessors recording the outcome measurements were blinded to the group allocations.

### 2.2. Participants

Male and female patients with low-risk and age ≥ 18 years, who had experienced an acute myocardial infarction or angina pectoris, had undergone revascularization (percutaneous transluminal coronary angioplasty or coronary artery bypass grafting), or suffered from coronary heart disease as documented by angiography, up to one year prior to the enrolment in the study were included. Exclusion criteria included unstable angina, atrial fibrillation, cardiac implantable electronic devices, complex ventricular arrhythmias, uncontrolled hypertension, and conditions limiting participation in exercise training and/or symptom-limited cardiopulmonary exercise test (CPET) at PRE. Finally, twenty-one men and two women were included. All participants were untrained and maintained their medication regimen throughout the intervention.

### 2.3. Measurements

#### 2.3.1. At Home Day-to-Day Heart Rate Variability Assessment

All patients were instructed to assess their HRV in the morning at home for 10 weeks. The HRV recordings were attained via a previously validated, photoplethysmography smartphone application (HRV4Ttraining) [33]. HRV assessments were carried out at rest, as patients lay supine for 90 s with spontaneous breathing in a semi-dark room, and the last 60 s were captured. The validity of ultra-short-term HRV indices (i.e., 60 s) has been previously reported, allowing the assessment of daily HRV [34,35]. Patients were asked to avoid talking or moving throughout the assessment. The smartphone application automatically deleted measurement errors and ectopic beats. The record was discarded and repeated immediately in case of erroneous signals.

Daily RMSSD values measured at home served two purposes: (1) as an outcome variable, where they were used to determine the pre- and post-intervention PNS status using the mean RMSSD of the days of the assessment weeks (i.e., weekly averaged RMSSD) (Figure 1) and; (2) as a decision-making variable, where daily RMSSD was used to prescribe training sessions during the TP for the HRV-G, as explained in depth in the subsection entitled “Exercise training programs”.

#### 2.3.2. Laboratory-Based Heart Rate Variability Assessment

Each patient underwent HRV assessment on the day of the CPET at the same hour (7 to 9 a.m.) on repeated testing (i.e., PRE and POST) to avoid bias due to circadian rhythms. Before the assessment, patients were familiarized with the material and protocol to increase the validity of the measurement [36]. Patients were advised to avoid eating or drinking anything eight hours before the assessment and not to perform any type of exercise for at least 48 h. A Polar H7 chest strap (Polar Electro OY, Kempele, Finland) and Elite HRV app [37] were used to capture HRV measurements. The assessments were performed with patients in a supine position in a quiet room with an average temperature of 22 °C. Patients were informed to avoid talking and falling asleep, controlling their breathing pace to 12 breaths per min. The length of the recording was 20 min, and the last 5 min was selected to calculate isolated (i.e., single value) vagal-related HRV indices. Kubios HRV Software 2.0 for Windows (The Biomedical Signal Analysis Group, Kuopio, Finland) was used to obtain isolated RMSSD, HF, and SD_1_ values, which were reported in the original scale. Data were detrended using a smoothness method. Fast Fourier transform was used to calculate power spectral density.

#### 2.3.3. Cardiopulmonary Exercise Test

CRF (i.e., VO_2_ max) was evaluated using a medically supervised maximal graded cycle ergometer exercise test (Excite Bike Med, Technogym, Cesana, Italy). Systolic blood pressure (SBP) and diastolic blood pressure (DBP) were assessed at resting condition before the CPET. Moreover, a 10 min resting assessment was performed in the seated position on the ergometer. Patients were instructed to avoid talking or moving throughout the resting assessment. The last 5 min was captured to obtain results at resting condition. Afterwards, a 3 min warm-up at 10 W was carried out, followed by a 1 min stage incremental exercise test to volitional exhaustion with 10 W work increments [38]. The pedaling frequency was fixed between 60 and 70 rpm. At the end of the test, a 3 min cooldown at 10 W was performed. 

Respiratory gas exchange was measured by MasterScreen CPX (Jaeger, Hoechberg, Germany) and HR was monitored continuously using a 12-lead electrocardiogram (Jaeger, Hoechberg, Germany) throughout the test. The plateau in oxygen uptake (VO_2_) was used to determine whether VO_2_ max had been reached [39]. Moreover, when the plateau did not appear, a respiratory exchange ratio (RER) > 1.10 was also used [40]. Breath-by-breath gas exchange measurements allowed the determination of VO_2_ and carbon dioxide production (VCO_2_). These data were averaged every 15 s. The VO_2_ max was defined as the highest VO_2_ mean value measured at the end of the test. First ventilatory threshold (VT1) and second ventilatory threshold (VT2) were analyzed blindly using the ventilatory equivalents method [41]. Variables were obtained at resting condition (i.e., VO_2_, HR, SBP, and DBP), VT2 (i.e., VO_2_, workload, and HR), and exercise peak (i.e., VO_2_ max, workload, and HR peak). VO_2_ max was included as the primary endpoint, while the remaining variables were included as secondary outcomes. HRR was measured in the seated position during the cooldown and was defined as the reduction in HR from the rate at peak exercise to the rate at 1 min (HRR 1 min) and 2 min (HRR 2 min) of the recovery period. These HRR indices were selected because they have been widely used in the literature, and there is evidence demonstrating their association with mortality risk [3].

#### 2.3.4. Secondary Outcomes

A detailed description of other analyzed secondary outcomes (i.e., body composition, blood analysis, quality of life, and dietary intake), as well as outcome data, is available in the Supplementary material (i.e., cardiopulmonary exercise test variables [Table S1], body composition [Table S2], blood analysis [Table S3], quality of life [Table S4], and dietary intake [Table S5]).

### 2.4. Exercise Training Programs

**Figure 2:**
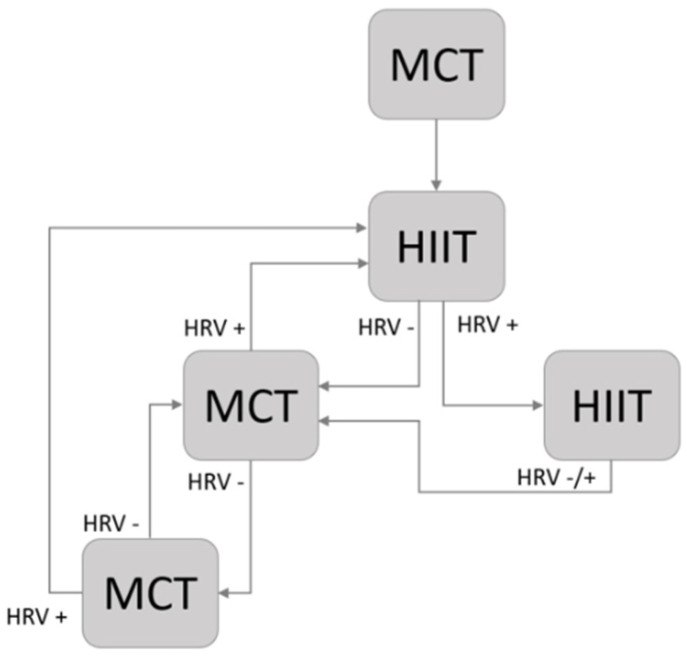
HRV-guided training schema. HRV−, LnRMSSD7-day fell outside SWC; HRV+, LnRMSSD7-day fell inside SWC; MCT, moderate continuous training; HIIT, high-intensity interval training.

During the two weeks of the BP, all the patients carried out two low-intensity sessions per week (four sessions) to familiarize themselves with the cycle ergometer and obtain a baseline HRV measurement. The length of the familiarization sessions ranged from 20 to 30 min. Throughout the TP, an individualized training program was prescribed according to the workload at VT1 (WVT1) and workload at VT2 (WVT2) derived from the CPET at PRE. The intensity of moderate continuous training (MCT) sessions ranged between WVT1 and WVT2, and the session length increased from 30 to 40 min. High-intensity interval training (HIIT) sessions consisted of four bouts of 4 min above WVT2 with 4 min of active recovery below WVT1. Each session started and finished with 5 min of warm-up and cooldown below WVT1. The training frequency in both groups was three times per week (18 sessions). The first session was always in the form of MCT, regardless of the training group. The frequency of HIIT sessions in the PRED-G increased every two weeks (1–2 weeks: 3 MCT/0 HIIT; 3–4 weeks: 2 MCT/1 HIIT; 5–6 weeks: 1 MCT/2 HIIT). The patients allocated in the HRV-G carried out MCT or HIIT sessions based on their daily HRV assessment following a decision schema (Figure 2). Patients allocated in the HRV-G did not accumulate more than two consecutive HIIT exercise sessions. All training sessions were performed under the supervision of qualified instructors. 

RMSSD was selected as the vagal-related HRV index to carry out training prescription since previous studies have reported that it reflects the PNS status better than other indices [29,42]. In addition, only for this purpose, the RMSSD data were logarithmically transformed to correct skewness (LnRMSSD). Subsequently, a 7-day rolling average of RMSSD (LnRMSSD7-day) was calculated to carry out training prescription in patients allocated to the HRV-G [43]. During the first three weeks of the study protocol (BP and PRE), the SWC of LnRMSSD was calculated as mean ± 0.5 × standard deviation (*SD*) [43,44] (SWC_1_). SWC_1_ was used to prescribe exercise training for the first three weeks of the TP in HRV-G patients. SWC was updated after the first three weeks of TP (SWC_2_) and used to carry out training prescription for the remaining TP weeks (Figure 1), as previous studies have reported a relationship between ANS status and performance [45].

### 2.5. Statistical Analyses

The Shapiro–Wilk test and the Levene test were used to test the normality of the data and the equality of the group variances (homoscedasticity), respectively. Furthermore, the normal distribution of the data was graphically verified by box plot and Q–Q graphs. Categorical variables are presented as number of cases (percentage). Normally distributed continuous variables are reported as mean ± *SD*, and those that were non-normally distributed are reported as median (25th and 75th percentiles). Percentiles were calculated by the weighted average method. The Student’s independent *t*-test, the Mann–Whitney *U* test, and the Fisher’s exact test were used for between-group comparisons in normally distributed continuous variables, non-normally distributed continuous variables, and categorical variables, respectively. The Student’s paired *t* test and the Wilcoxon’s signed-rank test were carried out for within-group comparisons in normally and non-normally distributed variables, respectively. Any changes in the outcomes following the intervention were quantified by subtracting the pre-intervention values from the post-intervention values, including 95% confidence interval (CI) for the mean or median change based on the normality assumption [46]. All analyses were considered statistically significant at a critical level of *p* ≤ 0.050. Within-group comparisons, regardless of the training group, were carried out if between-group differences in changes at follow-up did not reach statistical significance (*p* > 0.050). The intention-to-treat principle was applied in the sense that data were analyzed for all randomized patients for whom post-intervention data were available. G*Power was used to estimate the required sample size a priori (*α* = 0.050, 1 − *β* = 0.800), which yielded a total of 24 participants with an effect size of *d* = 1.2, based on the results of a previous study about the effect of HRV-guided training on resting vagal-related HRV indices (i.e., weekly averaged RMSSD) [27]. STATA software was used to perform statistical analyses (version 16.0; Stata Corp LLC, College Station, TX, USA).

## 3. Results

**Figure 3:**
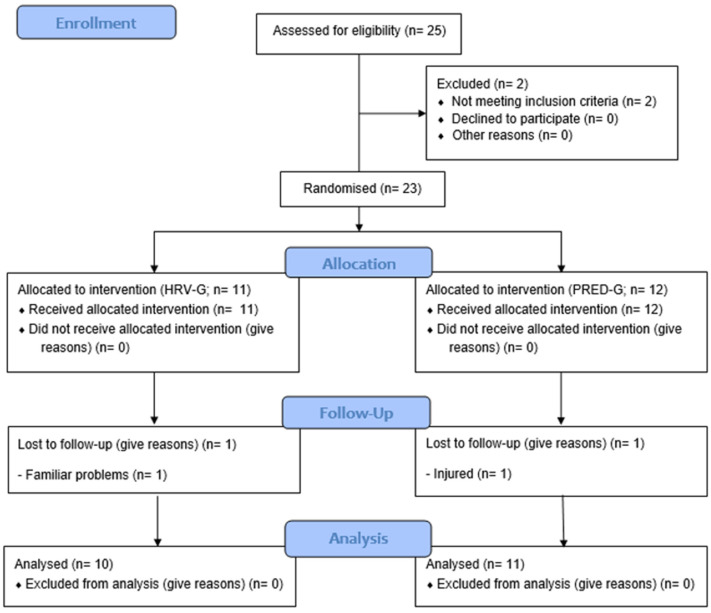
CONSORT 2010 flow diagram. HRV-G, heart rate variability-guided training group; PRED-G, predefined training group.

Twenty-five patients were recruited to participate in this study. After baseline assessments, two patients were not allocated because they showed exercise limitations. Therefore, 23 patients were randomized to the HRV-G (*n* = 11) or PRED-G (*n* = 12), of which two patients (9%, one in the HRV-G and one in the PRED-G) dropped out of the study. Thus, a total of 21 patients, comprising 10 patients in the HRV-G and 11 patients in the PRED-G, completed a six-week CR program, carrying out 18 exercise sessions (100% compliance) (Figure 3). No events happened throughout the intervention. Patients allocated to the PRED-G performed six HIIT sessions throughout the TP (based on the predefined training program), while the mean ± *SD* HIIT sessions in the HRV-G was 6.9 ± 1.7 (min–max: four–nine sessions). Demographic and clinical characteristics of the study patients at baseline are reported in Table 1. It should be pointed out that only two females were included in the study (one in the HRV-G and one in the PRED-G). Moreover, the proportion of diabetic patients was statistically significantly lower (*p* = 0.035) in the HRV-G (0.0%) than in the PRED-G (45.5%), while no statistically significant between-group differences (*p* > 0.050) were found in the remaining baseline characteristics. 

### Mortality Predictors

Pre- and post-intervention values, and changes in HR-based indices (i.e., vagal-related HRV indices, resting HR, and HRR indices) and VO_2_ max are reported in Table 2. There were no statistically significant between-group differences in any of the analyzed variables at baseline (*p* > 0.050). A statistically significant between-group difference was found for the change at follow-up in the weekly averaged RMSSD (*p* = 0.034). A statistically significant increase in weekly averaged RMSSD after HRV-guided training (*p* = 0.039; mean change (MC) = 7.57 [95% CI = 0.48–14.64] ms) was found, while no significant change in weekly averaged RMSSD was found after predefined training (*p* = 0.416; MC = −2.79 [95% CI = −10.13–4.55] ms). No statistically significant differences were found in the remaining analyzed variables (*p* > 0.050). Regardless of the training prescription method, statistically significant changes in resting HR (*p* = 0.001; MC = −4.10 [95% CI = −6.37–−1.82] beats per minute (bpm)), HRR 2 min (*p* = 0.010; MC = 4.33 [95% CI = 1.15–7.52] bpm), and VO_2_ max (*p* < 0.001; MC = 3.04 [95% CI = 1.70–4.37] mL·kg^−1^·min^−1^) were found.

## 4. Discussion

This research aimed to investigate whether HRV-guided training improves mortality predictors (i.e., HR-based indices and VO_2_ max) to a greater extent than predefined training in patients with CAD. Additionally, if no differences were found between HRV-guided training and predefined training, the effects of exercise-based CR, regardless of the training prescription method, on mortality predictors were also investigated. In line with our hypothesis, we found that HRV-guided training is superior to predefined training in improving PNS modulation, assessed by weekly averaged RMSSD. On the other hand, in contrast to our hypothesis, HRV-guided training is not superior to predefined training in improving other mortality predictors (i.e., resting HR, HRR indices, and VO_2_ max). Interestingly, there is evidence that shows that predefined exercise-based CR programs enhance HRR indices and VO_2_ max [9,18], while the training-induced effect on vagal-related HRV indices seems to be more controversial [18]. The efficacy of predefined exercise-based CR programs on these mortality predictors may help to explain the lack of superiority of HRV-guided training for improving HRR indices and VO_2_ max. Nonetheless, HRV-guided training seems to be a suitable training prescription method to overcome the limitations of predefined exercise-based CR programs for enhancing vagal-related HRV indices. Regardless of the training prescription method, the findings of this study showed that exercise-based CR enhances resting HR, HRR 2 min, and VO_2_ max. 

To the best of our knowledge, this is the first study to examine the superiority of HRV-guided training in improving mortality predictors (i.e., HR-based indices and VO_2_ max) compared to predefined training in CAD patients. As has been mentioned above, previous studies have been carried out in CHF patients, sedentary healthy people, and endurance-trained athletes, showing high methodological heterogeneity for applying HRV-guided training, such as RMSSD [26,28,44] vs. HF [30], isolated HRV values [27,28,30] vs. averaged HRV values [26,44], standing position [27,28,30] vs. supine position [26,44] or morning [26,27,30,44] vs. afternoon/evening measurements [28]. We decided to use a 7-day rolling average of RMSSD values as their sensitivity to training status is higher than isolated HRV values [29]. Additionally, the patients in this study carried out daily HRV assessment in the supine position in the morning. Assessments of HRV performed in the morning allow daily stressors to be easily controlled [30]. Moreover, there is evidence that the standing position is more suitable than the supine position in endurance-trained athletes to avoid saturation of acetylcholine receptors due to heightened PNS tone (i.e., bradycardia) [42], which diminishes vagal-related HRV indices [47]. Nonetheless, enhanced PNS tone is not common in CAD patients. Future studies carried out in CAD patients should test the influence of these methodological approaches on the applicability of HRV-guided training for enhancing the effectiveness of exercise-based CR on mortality predictors. 

The findings of this study showed that HRV-guided training is superior to predefined training in enhancing resting PNS modulation, assessed by weekly averaged RMSSD. It should be pointed out that, although no statistical differences were found, the number of patients using HRV-modifying drugs (i.e., angiotensin II receptor blocker and calcium-channel blockers) was higher in the PRED-G than in the HRV-G (see Table 1), which may mitigate the magnitude of the differences in favor of HRV-guided training for increasing vagal-related HRV indices [48]. In contrast, no differences were found between both training prescription methods when isolated vagal-related HRV indices (i.e., RMSSD, HF, or SD_1_) were used for inferring PNS modulation. These controversial findings could be due to the influence of methodological issues on the sensitivity of vagal-related HRV indices to detect PNS status, such as the influence of breathing patterns on HRV values and the natural day-to-day HRV lability. In this regard, Buchheit [42] and Saboul et al. [49] reported that RMSSD has lower sensitivity to breathing patterns than HF, while Plews, Laursen, Le Meur, Hausswirth, Kilding and Buchheit [20] found that averaged HRV values appear to be a better method for assessing training-induced PNS adaptation compared to isolated HRV values due to the reduction in day-to-day HRV lability. Along the same lines, Le Meur et al. [50] revealed that isolated HRV measurements may not detect training-induced PNS modulation in athletes after an overload training period, while Manresa-Rocamora, Flatt, Casanova-Lizón, Ballester-Ferrer, Sarabia, Vera-Garcia and Moya-Ramón [19] reported that averaged HRV values provide the best evidence of PNS modulation. This evidence supports our idea that averaged RMSSD values may be a more suitable vagal-related HRV index to obtain information on the PNS modulation. Nonetheless, the influence of methodological issues (i.e., isolated vs. averaged HRV values) on the sensitivity of vagal-related HRV indices to detect PNS hyperactivity in patients with CAD should be addressed in future studies. In line with the findings of the present study, da Silva, Ferraro, Adamo and Machado [28] and Kiviniemi, Hautala, Kinnunen, Nissilä, Virtanen, Karjalainen and Tulppo [27] only found an increase in resting PNS modulation (i.e., RMSSD or SD_1_) in the HRV-guided training groups, while no changes were found in the predefined training groups. Therefore, based on our findings and this previous evidence, as we hypothesized, it seems that HRV-guided training is superior to predefined training in improving resting PNS modulation in patients with CAD, which could help to reduce mortality rates in these patients [5]. Nevertheless, weekly averaged RMSSD should be used to increase the sensitivity of HRV measurements to detect CR-induced PNS adaptation.

**Figure 4:**
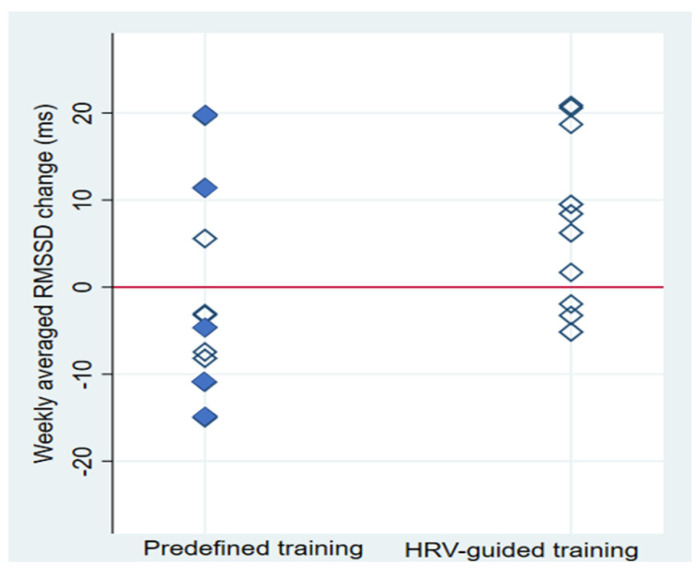
Individual changes in the weekly averaged root-mean-square difference of successive normal R-R intervals (RMSSD) based on the training prescription group. HRV, heart rate variability; filled and empty diamonds denote diabetic and non-diabetic patients, respectively.

As has been commented previously, the individual response to the same exercise-based CR program varies among patients. Individual changes in weekly averaged RMSSD values after exercise-based CR, based on the training prescription group, are shown in Figure 4. Most of the patients who carried out HRV-guided training increased weekly averaged RMSSD values (70%), while individual changes in this vagal-related HRV index in response to predefined training were more heterogeneous (27% showed increased weekly averaged RMSSD values). It should be taken into account that all diabetic patients were randomly allocated in the PRED-G, an issue that could potentially influence our findings. Nonetheless, as depicted in Figure 4, some diabetic patients also increased their weekly averaged RMSSD, allowing us to rule out this comorbidity as a threat to the validity of our results. This finding is in line with those previously reported by Kiviniemi, Hautala, Kinnunen and Tulppo [30] and Javaloyes, Sarabia, Lamberts, Plews and Moya-Ramon [32] about the effect of HRV-guided training on CRF and endurance performance, respectively. In this regard, there is evidence that shows the influence of the PNS status, which is the physiological criterion used to carry out HRV-guided training, on the response to aerobic training [24], which may explain the lower heterogeneity in response to HRV-guided training compared to predefined training. Nonetheless, the heterogeneity in response to training based on the training prescription method used requires future investigation.

Regarding other HR-based indices, in contrast to our hypothesis, there was no increase in HRR 1 min after exercise-based CR. There is evidence that exercise-based CR is a non-pharmacological treatment for enhancing HRR 1 min, which is primarily PNS-mediated [51]. For instance, Manresa-Rocamora, Ribeiro, Sarabia, Íbias, Oliveira, Vera-García and Moya-Ramón [18] reported an increase of 5.35 (95% CI = 4.08–6.61) bpm in HRR 1 min after exercise-based CR compared to usual care. Nonetheless, HRR 1 min at baseline in the studies included in this latter meta-analysis (mean ± *SD*: 10.88 ± 5.17 bpm) was lower than HRR 1 min at pre-intervention shown by the participants (mean ± *SD*: 20.00 ± 9.90 bpm), even though this HR-based index was measured during an active recovery period, which diminishes HRR 1 min [52,53]. Therefore, the controversial finding of the present study could be due to the high HRR 1 min showed by the included patients at baseline, which could limit the possibility of improving PNS reactivation in these patients after exercise-based CR. On the other hand, the results of the present study showed an increase of 4.33 (95% CI = 1.15–7.52) bpm in HRR 2 min after exercise-based CR. The recovery of HR at 2 min after exercise is affected by PNS reactivation and SNS withdrawal, as reported by Perini, Orizio, Comandè, Castellano, Beschi and Veicsteinas [17], who found a plasma norepinephrine concentration reduction during the second minute of the recovery. In line with the present findings, Wang et al. [54] found an enhancement in HRR 2 min after a four-week multimedia CR program compared to usual care in cardiac patients. The improvement in HRR 2 min after exercise-based CR could also be due to a faster clearance of metabolites, which diminishes metaboreflex stimulation and, therefore, increases HRR 2 min [55]. The enhancement of HRR 2 min also could help to reduce mortality in CAD patients [56]. Finally, this study also showed that exercise-based CR diminishes resting HR, which reflects an increase in the PNS tone [57]. 

Finally, the current study showed that HRV-guided training is not superior to predefined training in improving VO_2_ max, which is in line with the results of previous systematic reviews and meta-analyses that included studies carried out with healthy people and endurance-training athletes [58,59]. As has been previously commented, predefined exercise-based CR enhances VO_2_ max [9], which may explain the lack of superiority of HRV-guided training in improving this mortality predictor compared to predefined training. In contrast to the finding of the present study, Behrens, Hottenrott, Weippert, Montanus, Kreuzfeld, Rieger, Lübke, Werdan and Stoll [31], who performed a four-week exercise-based CR program, found that HRV-guided training enhances VO_2_ max in CHF patients, while no changes were observed after predefined training. Nonetheless, there is evidence that shows that the effect of predefined exercise-based CR programs on VO_2_ max in patients with CHF is lower in shorter interventions [60], while no influence of the intervention length on the training-induced effect on VO_2_ max has been reported in patients with CAD [9,61]. Therefore, it seems that in CHF patients, whose hallmark is exercise intolerance, HRV-guided training could induce faster CRF adaptations than predefined training. Based on these controversial findings, future studies should test the influence of training characteristics (e.g., intervention length, training frequency) on the applicability of HRV-guided training for improving mortality predictors. Regardless of the training prescription method, the present study showed an improvement of 3.04 (95% CI = 1.70–4.37) mL·kg^−1^·min^−1^ in VO_2_ max after a six-week CR program, which is in line with previously reported results [9].

## 5. Strengths and Limitations

This randomized, double-blind study is the first to test the superiority of HRV-guided training for improving PNS function and CRF compared to predefined training in patients with CAD. All sessions have been carried out under the supervision of qualified instructors on previously established weekdays (Monday, Wednesday, and Friday) at the same time of the day, which allows better control of the training sessions and avoids variations from the exercise session to the following HRV assessment. Moreover, weekly averaged RMSSD was also included as a vagal-related HRV index, which increases the validity of our findings on the training-induced effect on vagal-related HRV indices. Nevertheless, there are also some limitations that should be highlighted. No patients were included in a usual care group, which does not allow us to discard the influence of confounding factors against the validity of our results. However, the main objective of this study was to compare the effects of both training prescription methods on improving the analyzed variables. Most of the patients included in the present study were males (90%). Therefore, these findings should be limited to male CAD patients, and future studies should test the efficacy of HRV-guided training in female CAD patients. Finally, day-to-day HRV assessments were performed at home since it is more convenient for the patients. For this reason, to increase the applicability of this model, it was decided to allow patients to breathe spontaneously, which did not allow us to analyze the effect of exercise-based CR on averaged HF values, since this latter variable is affected by breathing patterns.

## 6. Conclusions

According to the findings of the present study, HRV-guided training is superior to predefined training in improving resting PNS modulation, assessed by weekly averaged RMSSD, in patients with CAD. However, it should be highlighted that contradictory findings were found based on the vagal-related HRV indices used, which could be due to the influence of methodological issues on the sensitivity of isolated vagal-related HRV indices for inferring PNS modulation. Moreover, HRV-guided training is not superior to predefined training for enhancing other mortality predictors (i.e., resting HR, HRR indices, and VO_2_ max). However, improved PNS modulation is likely relevant given the independent association between vagal-related HRV indices and cardiovascular morbidity and mortality. Regardless of the training prescription method used to prescribe aerobic training, exercise-based CR enhances resting HR, HRR 2 min, and VO_2_ max.

## Figures and Tables

**Table 1:** Demographic and clinical characteristics of the study patients at baseline.

	PRED-G (*n* = 11)	HRV-G (*n* = 10)	*p*
Age (years)	59.2 ± 6.9	56.9 ± 5.6	0.418
Sex (male)	10 (90.9)	9 (90.0)	0.999
Body weight (kg)	78.2 ± 7.7	77.1 ± 15.5	0.852
Body mass index (kg/m^2^)	29.1 (27.7, 29.7)	26.8 (26.0, 30.6)	0.569
Wait time (days)	215.4 ± 84.9	169.2 ± 57.8	0.166
No. of infarcted patients	7 (63.6)	7 (70.0)	0.999
Site of infarction (anterior)	7 (63.6)	5 (50.0)	0.670
No. of vessels involved (1 vessel)	8 (72.7)	7 (70.0)	0.999
No. of events (first)	10 (90.9)	9 (90.0)	0.999
No. PTCA intervention surgery	10 (90.9)	9 (90.0)	0.999
Diabetes mellitus	5 (45.5)	0 (0.0)	0.035 *
Hypertension	5 (45.5)	3 (30.0)	0.659
Smoker	5 (45.5)	4 (40.0)	0.999
Hyperlipidemia	6 (54.5)	5 (50.0)	0.999
Overweight/obesity	3 (27.3)	1 (10.0)	0.586
Family history	2 (18.2)	3 (30.0)	0.635
Personal history	2 (18.2)	1 (10.0)	0.999
b-Blockers	9 (81.8)	9 (90.0)	0.999
ACE inhibitors	4 (36.4)	5 (50.0)	0.670
Antiplatelets	11 (100)	9 (90.0)	0.476
Diuretics	4 (36.4)	1 (10.0)	0.311
Nitrates	11 (100)	10 (100)	N/A
ARBs	6 (54.5)	2 (20.0)	0.183
Calcium-channel blockers	3 (27.3)	0 (0.0)	0.214
Lipid-lowering drugs	11 (100)	10 (100)	N/A

ACE, angiotensin-converting enzyme; ARB, angiotensin II receptor blocker; HRV-G, heart rate variability-guided training group; *p*, probability associated with the statistic; PRED-G, predefined training group; PTCA, percutaneous transluminal coronary angioplasty; wait time: time from procedure or event to start of exercise-based cardiac rehabilitation. Data are delivered as number of cases (percentage), mean ± *SD* or median (25th and 75th percentiles). *p* values refer to differences between the randomized groups at baseline in patients completing the intervention; * denotes *p* ≤ 0.050.

**Table 2:** Effect of exercise-based cardiac rehabilitation on mortality predictors.

		Based on the Training Group (PRED-G, *n* = 11; HRV-G, *n* = 10)					All Patients (*n* = 21)			
Variable	Group	Pre	Post	*p ^A^*	Change (95% CI)	*p ^B^*	Pre	Post	*p ^A^*	Change (95% CI)
Isolated RMSSD (ms)	PRED-G	28.4 ± 11.8	29.4 ± 10.2	0.774	1.07 (−7.12–9.26) −2.79 (−9.90–4.32)	0.431	35.1 ± 25.7	34.3 ± 21.8	0.721	−0.86 (−5.83–4.11)
	HRV-G	41.9 ± 34.0	39.1 ± 29.1	0.398						
Isolated HF (ms^2^)	PRED-G	272.0 (151.3, 663.0)	480.5 (188.5, 670.3)	0.846	−27.0 ^#^ (−225.2–425.4) −111.5 ^#^ (−892.3–43.8)	0.143	374.0 (175.3, 745.0)	357.5 (207.5, 649.8)	0.202	−66.00 ^#^ (−236.59–53.53)
	HRV-G	649.5 (190.3, 1269.0)	353.5 (197.5, 627.0)	0.065						
Isolated SD_1_ (ms)	PRED-G	20.1 ± 8.3	20.7 ± 7.1	0.824	0.61 (−5.42–6.64) −2.16 (−7.07–2.75)	0.430	24.9 ± 18.2	24.1 ± 15.4	0.654	−0.78 (−4.34–2.79)
	HRV-G	29.7 ± 24.1	27.5 ± 20.7	0.346						
Weekly averaged RMSSD (ms)	PRED-G	57.6 ± 20.0	54.8 ± 19.8	0.416	−2.79 (−10.13–4.55)	0.034 *	NA	NA	NA	NA
	HRV-G	49.7 ± 16.0	57.3 ± 18.3	0.039 *	7.57 (0.48–14.64)					
Resting HR (bpm)	PRED-G	64.7 ± 5.0	62.2 ± 7.3	0.068	−2.55 (−5.32–0.23) −5.80 (−9.74–−1.86)	0.140	63.9 ± 7.1	59.8 ± 9.1	0.001 *	−4.10 (−6.37–−1.82)
	HRV-G	63.0 ± 9.1	57.2 ± 10.6	0.009 *						
HRR 1 min (bpm)	PRED-G	17.6 ± 6.8	19.1 ± 5.7	0.108	1.45 (−0.38–3.29) 0.10 (−1.53–1.73)	0.235	20.0 ± 9.9	20.9 ± 9.1	0.163	0.81 (−0.36–1.98)
	HRV-G	22.7 ± 12.3	22.8 ± 11.8	0.893						
HRR 2 min (bpm)	PRED-G	28.4 ± 9.2	34.4 ± 7.3	0.014 *	6.00 (1.19–10.51) 1.00 (−2.35–7.38)	0.263	32.0 ± 12.6	36.3 ± 10.9	0.010 *	4.33 (1.15–7.52)
	HRV-G	36.0 ± 15.0	38.5 ± 14.0	0.300						
VO_2_ max (mL·kg^−1^·min^−1^)	PRED-G	25.0 ± 5.7	28.1 ± 6.0	0.005 *	3.16 (1.21–5.11)	0.851	24.9 ± 5.4	28.0 ± 6.0	<0.001 *	3.04 (1.70–4.37)
	HRV-G	24.9 ± 5.3	28.0 ± 6.4	0.017 *	2.91 (0.67–5.15)					

CI, confidence interval; HF, high frequency; HR, heart rate; HRR 1 min; heart rate recovery 1 min; HRR 2 min; heart rate recovery 2 min; HRV-G, heart rate variability-guided training group; NA, non-applicable; PRED-G, predefined training group; RMSSD, the root mean square of the differences between successive R-R intervals; SD_1_, the standard deviation of instantaneous beat-to-beat R-R interval variability; VO_2_ max, maximum oxygen uptake. Data at pre- and post-intervention are delivered as mean ± *SD* or median (25th and 75th percentiles); *p ^A^* and *p ^B^* values refer to within-group and between-group differences, respectively; * denotes *p* ≤ 0.050; ^#^ denotes median change instead of mean change.

## Data Availability

The dataset generated from the current study are available from the corresponding author on reasonable request.

## Supplementary Materials

The following are available online at https://www.mdpi.com/article/10.3390/ijerph191710463/s1, Table S1 Effect of exercise-based cardiac rehabilitation on the cardiopulmonary exercise test variables at exercise peak, second ventilatory threshold and resting condition; Table S2 Effect of exercise-based cardiac rehabilitation on the body composition; Table S3 Effect of exercise-based cardiac rehabilitation on the biochemical and hematology variables; Table S4 Effect of exercise-based cardiac rehabilitation on the quality of life; Table S5 Dietary intake. References [62,63,64,65,66,67,68] are cited in the supplementary materials.
