# Supplementary material for: Heart Rate Variability-Guided Training for Improving Mortality Predictors in Patients with Coronary Artery Disease
Source: Int J Environ Res Public Health. 2022 Aug 23;19(17):10463. doi: 10.3390/ijerph191710463 (PMC9518028; doi:10.3390/ijerph191710463)
Supplement: Supplementary file 1 [file ijerph-19-10463-s001.zip › ijerph-1860226-supplementary.pdf]

Supplementary Materials.

Supplemental methods

Secondary outcomes

Body composition

All anthropometrical variables were measured by a Level 2 anthropometrist certified by the International Society for the Advance of Kinanthropometry (ISAK) with an individual technical error of measurement (TEM) of 0.76–0.39% for skinfolds and of 0.12% for the remaining parameters. The errors were considered acceptable for ISAK standards (<7.5% for skinfolds and <1.5% for the remaining measurements). All measurements were made following the guidelines stated by ISAK [1]. The total body mass of each participant was measured in kilograms using a digital scale (Tanita, TBF 300 A, Tokyo, Japan), breadths with a Holtain bicondylar caliper (Holtain, UK), girths with a metallic non-extensible tape (Lufkin, USA), and skinfolds with a Holtain Tanner/Whitehouse skinfold caliper (Holtain, UK). The following four breadths were measured: humerus, wrist, femur, and ankle. Regarding girths, four were measured: relaxed arm, flexed and tensed arm, thigh, and medial calf. Finally, eight skinfolds were also measured: triceps, biceps, subscapular, ileocrestal, supraspinale, abdominal, thigh, and medial calf. Percentage of body fat mass was calculated using Durnin-Womersley equation [2]. Percentage of bone mass was calculated according to Martin's equation [3]. The percentage of muscle mass was calculated from Lee's equation [4]. In addition, the sums of the eight skinfolds were considered for fat content calculations.

Blood collection

Blood samples were obtained from the median cubital vein at PRE and POST after at least a 10-hour fast. Venipuncture (Höfer, Doering, Rumpold, Oldridge, & Benzer, 2006). Blood was centrifuged (2500 ×g for 15 min at 4 °C) to obtain serum or plasma, and frozen at −80 °C until further analysis. Measurement of glucose, urea, creatinine, uric acid, LDH, CK, sodium,

potassium, total cholesterol, HDL-C, LDL-C, triglycerides, haemoglobin A1c, platelet, red blood cells and haemoglobin was performed using standardized methods.

Quality of life

The MacNew heart disease health-related quality of life (HRQL) instrument was used as a disease-specific HRQL questionnaire [5]. MacNew consists of 27 items that fall into three domains (a 13-item physical limitations domain scale, a 14-item emotional function domain scale, and a 13-item social function domain scale). The maximum possible score in any domain is 7 (high HRQL) and the minimum is 1 (poor HRQL).

Dietary intake

Patients were indicated to follow the dietetic instructions they received during the time they stayed at the hospital. No personal diet was provided. Patients recorded their food and beverage consumption during 4 consecutive days, including Sunday, before and after the intervention. Data from records was analyzed using the ST-Nutrition software (Servitux, Elche, Spain). Macro- and micro-nutrient intakes were evaluated paying attention to the recommendations established by the Portfolio dietary pattern for the National Cholesterol Education Program (NECP) Step II [6]. Recommendations from FESNAD (Spanish Federation of Nutrition, Food and Dietetics Associations)-SEEDO (Spanish Association for the Study of Obesity) were considered as well, taking into account the strong relation of overweight and obesity as risk factors to develop cardiovascular disease [7]. Basal metabolic rate of participants was estimated according to FAO/WHO recommendations (http://www.fao.org/3/y5686e/y5686e07.htm#bm07). Physical activity level was expressed as a multiple of 24-h basal metabolic rate, considering 1.53 for light activity lifestyle.

Outcome data

Pre- and post-intervention values, and changes at follow-up in CPET variables, body composition, biochemical and hematological variables, and quality of life are reported in Tables

S1 – S4, respectively. There were no between-group statistically significant differences at baseline (p > .050). No between-group statistically significant differences were found for the change reached at follow-up in any of the secondary outcomes (p > .050). The effect of exercise-based CR on secondary outcomes are shown in the Supplementary digital material.

Regarding dietary intake, pre- and post-intervention values of the 4-day recording of energy, water, and macronutrients are shown in Table S5 (Supplementary digital material). There were no between-group statistically significant differences at pre- and post-intervention for any of the analyzed variables (p > .050). Within-group analyses showed a significant decrease at follow-up in eicosapentaenoic acid and docosahexaenoic acid for the PRED-G, and in cholesterol intakes for the HRV-G ($p \leq .050$).

Table S1 Effect of exercise-based cardiac rehabilitation on the cardiopulmonary exercise test variables at exercise peak, second ventilatory threshold and resting condition

		Based on the training group (PRED-G, n = 11; HRV-G, n = 10)					All patients (n = 21)			
Variable	Group	Pre	Post	p^A	Change (95% CI)	p^B	Pre	Post	p^A	Change (95% CI)
Workload (Watts)	PRED-G	173.0<br>(120.0, 203.0)	216.5<br>(145.0, 226.4)	.002*	28.50 [#]<br>(7.97 – 45.00)	.540	173.0<br>(123.0, 203.0)	211.5<br>(136.3, 236.8)	<.001*	27.50 [#]<br>(12.12 – 37.00)
	HRV-G	156.5<br>(124.5, 221.3)	198.8<br>(131.3, 239.1)	.020*	25.00 [#]<br>(8.96 – 36.35)					
HR peak (bpm)	PRED-G	141.3 ± 19.0	144.9 ± 21.2	.283	3.60<br>(-3.54 – 10.74)	.797	139 ± 18.2	143.5 ± 19.8	.057	4.15<br>(-0.14 – 8.44)
	HRV-G	137.4 ± 18.2	142.1 ± 19.3	.125	4.70<br>(-1.58 – 10.98)					
- Second ventilatory threshold										
Workload (Watts)	PRED-G	128.6 ± 32.0	147.5 ± 50.0	.043*	18.85<br>(0.77 – 36.93)	.465	124.2 ± 32.8	146.6 ± 43.9	<.001*	22.35<br>(12.65 – 32.05)
	HRV-G	119.8 ± 34.8	145.7 ± 39.6	<.001*	25.85<br>(14.74 – 36.95)					
HR (bpm)	PRED-G	120.0<br>(105.0, 126.0)	127.0<br>(108.0, 133.0)	.122	2.00 [#]<br>(-0.29 – 9.57)	.987	113.0<br>(101.5, 132.0)	123.0<br>(105.0, 133.5)	.038*	2.00 [#]<br>(0.01 – 7.55)
	HRV-G	109.0<br>(100.3, 134.0)	121.5<br>(100.0, 134.3)	.275	4.00 [#]<br>(-0.68 – 8.68)					
VO_2<br>($ml \cdot kg^{-1} \cdot min^{-1}$)	PRED-G	18.9 ± 4.7	23.5 ± 6.6	.003*	4.56<br>(1.97 – 7.15)	.432	19.4 ± 4.5	23.3 ± 6.3	<.001*	3.94<br>(2.32 – 5.55)
	HRV-G	19.9 ± 4.4	23.2 ± 6.4	.012*	3.31<br>(0.93 – 5.69)					
- Resting condition										
VO_2<br>($ml \cdot kg^{-1} \cdot min^{-1}$)	PRED-G	3.3 ± 0.4	3.7 ± 0.4	.003*	0.36<br>(0.16 – 0.56)	.135	3.4 ± 0.5	3.7 ± 0.6	.007*	0.24<br>(0.07 – 0.41)
	HRV-G	3.5 ± 0.6	3.7 ± 0.7	.372	0.12<br>(-0.17 – 0.41)					
SBP<br>(mmHg)	PRED-G	133.0 ± 13.9	129.2 ± 15.1	.393	-3.82<br>(-13.34 – 5.70)	.767	129.3 ± 15.7	126.3 ± 14.8	.293	-3.00<br>(-8.80 – 2.80)
	HRV-G	125.2 ± 17.3	123.1 ± 14.6	.584	-2.10<br>(-10.46 – 6.26)					
DBP<br>(mmHg)	PRED-G	80.5 ± 10.4	81.7 ± 8.7	.719	1.18<br>(-5.93 – 8.30)	.324	81.1 ± 9.3	80.1 ± 10.0	.655	-1.05<br>(-5.86 – 3.77)
	HRV-G	81.8 ± 8.4	78.3 ± 11.5	.322	-3.50<br>(-11.05 – 4.04)					

CI, confidence interval; *DBP*, diastolic blood pressure; *HR*, heart rate; *HRV-G*, heart rate variability-guided training group; *PRED-G*, predefined training group; *SBP*, systolic blood pressure; *VO_2*, oxygen uptake

Data at pre- and post-intervention are delivered as mean ± *SD* or median (25th and 75th percentiles); p^A and p^B values refer to within-group and between-group differences, respectively; * denotes $p \leq .050$; [#] denotes median change instead of mean change

Table S2 Effect of exercise-based cardiac rehabilitation on the body composition

		Based on the training group (PRED-G, n = 11; HRV-G, n = 10)					All patients (n = 21)			
Variable	Group	Pre	Post	p^A	Change (95% CI)	p^B	Pre	Post	p^A	Change (95% CI)
Weight (kg)	PRED-G	78.2 ± 7.7	78.2 ± 8.1	.962	0.02 (-0.82 – 0.85)	.560	76.9 ± 11.5	76.8 ± 12.2	.635	-0.14 (-0.72 – 0.45)
	HRV-G	75.4 ± 15.4	75.1 ± 16.3	.482	-0.32 (-1.33 – 0.69)					
BMI ($kg \cdot m^{-2}$)	PRED-G	28.05 ± 2.42	28.22 ± 2.41	.344	0.16 (-0.20 – 0.53)	.442	27.60 ± 3.42	27.67 ± 3.59	.598	0.07 (-0.20 – 0.34)
	HRV-G	27.03 ± 4.45	26.99 ± 4.74	.840	-0.04 (-0.54 – 0.45)					
∑8Sk	PRED-G	125.0 ± 23.9	119.3 ± 27.6	.009*	-5.68 (-9.63 – -1.73)	.581	121.6 ± 35.0	116.7 ± 37.4	.005*	-4.90 (-8.07 – -1.72)
	HRV-G	117.5 ± 46.4	113.6 ± 48.5	.180	-3.93 (-10.10 – 2.24)					
Waist-hip ratio	PRED-G	0.99 ± 0.06	0.98 ± 0.06	.124	-0.01 (-0.03 – 0.00)	.447	0.98 ± 0.08	0.97 ± 0.08	.151	-0.01 (-0.02 – 0.00)
	HRV-G	0.96 ± 0.09	0.95 ± 0.10	.701	-0.00 (-0.02 – 0.02)					
Percent body fat (%)	PRED-G	31.1 ± 4.6	30.6 ± 5.1	.129	-0.59 (-1.38 – 0.20)	.655	30.1 ± 5.4	29.3 ± 6.0	.022*	-0.71 (-1.30 – -0.11)
	HRV-G	28.7 ± 6.3	27.9 ± 6.9	.111	-0.85 (-1.95 – 0.24)					
Percent muscle mass (%)	PRED-G	36.5 (34.5, 37.9)	36.3 (33.0, 38.8)	.965	-0.07 # (-1.17 – 0.50)	.304	36.5 (35.1, 38.0)	36.8 (34.7, 39.0)	.615	0.20 # (-0.14 – 0.44)
	HRV-G	36.6 (35.5, 38.8)	37.4 (35.3, 39.1)	.359	0.27 # (-0.53 – 0.93)					

BMI, body mass index; *CI*, confidence interval; *HRV-G*, heart rate variability-guided training group; *PRED-G*, predefined training group; ∑8Sk, the sum of subscapular, triceps, biceps, ileocrestal, supraspinale, abdominal, thigh, and calf skinfolds

Data at pre- and post-intervention are delivered as mean ± *SD* or median (25th and 75th percentiles); p^A and p^B values refer to within-group and between-group differences, respectively; * denotes $p \leq .050$; # denotes median change instead of mean change

Table S3 Effect of exercise-based cardiac rehabilitation on the biochemical and hematology variables

		Based on the training group (PRED-G, n = 11; HRV-G, n = 10)					All patients (n = 21)			
Variable	Group	Pre	Post	p^A	Change (95% CI)	p^B	Pre	Post	p^A	Change (95% CI)
Glucose ($mg \cdot dL^{-1}$)	PRED-G	111.7 ± 15.0	112.5 ± 14.5	.838	0.73 (-6.98 – 8.43)	.240	107.6 ± 15.6	111.7 ± 17.6	.203	4.10 (-2.40 – 10.60)
	HRV-G	102.6 ± 15.6	110.8 ± 21.7	.164	8.22 (-4.14 – 20.58)					
Urea ($mg \cdot dL^{-1}$)	PRED-G	41.0 (34.0, 52.0)	39.0 (35.0, 51.0)	.333	-1.00 [#] (-10.29 – 5.57)	.500	41.0 (35.5, 50.5)	39.0 (35.5, 48.0)	.615	-1.00 [#] (-5.00 – 2.55)
	HRV-G	40.5 (36.3, 50.0)	40.5 (35.8, 47.3)	.999	0.00 [#] (-5.00 – 5.70)					
Creatinine ($mg \cdot dL^{-1}$)	PRED-G	1.0 (0.9, 1.0)	1.0 (0.8, 1.1)	.999	-0.01 [#] (-0.07 – 0.07)	.117	0.9 (0.8, 1.0)	0.9 (0.8, 1.0)	.316	0.03 [#] (-0.02 – 0.11)
	HRV-G	0.9 (0.7, 1.0)	0.9 (0.8, 1.0)	.168	0.10 [#] (-0.05 – 0.18)					
Uric acid ($mg \cdot dL^{-1}$)	PRED-G	5.3 (4.6, 5.9)	4.9 (4.5, 6.4)	.562	0.10 [#] (-0.66 – 0.33)	.499	5.1 (4.5, 6.6)	5.2 (4.4, 6.7)	.999	0.10 [#] (-0.51 – 0.35)
	HRV-G	4.9 (4.1, 6.8)	5.9 (4.1, 7.1)	.625	0.10 [#] (-0.67 – 0.90)					
LDH ($IU \cdot L^{-1}$)	PRED-G	326.0 (292.0, 368.0)	338.0 (309.0, 378.0)	.054	15.00 [#] (-3.16 – 35.59)	.877	317.0 (270.0, 362.5)	334.0 (288.5, 368.5)	.069	15.00 [#] (-1.10 – 32.10)
	HRV-G	293.0 (154.5, 360.8)	319.5 (277.0, 369.0)	.420	18.00 [#] (-46.40 – 195.73)					
CK ($IU \cdot L^{-1}$)	PRED-G	98.0 (84.0, 253.0)	115.0 (77.0, 180.0)	.700	8.00 [#] (-34.74 – 48.84)	.876	98.0 (76.0, 156.5)	100.0 (63.0, 162.5)	.702	10.00 [#] (-21.10 – 23.20)
	HRV-G	77.5 (53.0, 135.5)	100.0 (65.8, 145.8)	.902	12.00 [#] (-34.16 – 88.50)					
Sodium	PRED-G	142.0 (140.0, 143.0)	142.0 (139.0, 144.0)	.654	1.00 [#] (-3.29 – 4.00)	.353	141.0 (139.5, 143.0)	142.0 (142.0, 144.0)	.101	2.00 (-0.88 – 2.88)
	HRV-G	140.5 (139.0, 143.0)	144.0 (142.0, 144.0)	.027*	2.00 [#] (0.08 – 4.77)					
Potassium	PRED-G	4.7 (4.4, 4.7)	4.5 (4.4, 4.6)	.812	0.10 [#] (-0.46 – 0.30)	.358	4.6 (4.4, 4.9)	4.5 (4.4, 4.7)	.240	-0.05 [#] (-0.39 – 0.20)
	HRV-G	4.6 (4.4, 5.1)	4.5 (4.3, 4.7)	.203	-0.10 [#] (-0.59 – 0.19)					

Table S3 Continued

		Based on the training group (PRED-G, n = 11; HRV-G, n = 10)					All patients (n = 21)			
Variable	Group	Pre	Post	p^A	Change (95% CI)	p^B	Pre	Post	p^A	Change (95% CI)
Cholesterol (mg·dL-1)	PRED-G	129.2 ± 36.0	128.2 ± 27.3	.827	-1.00 (-10.93 – 8.93)	.339	135.6 ± 122.4	130.9 ± 25.7	.250	-4.76 (-13.14 – 3.62)
	HRV-G	142.7 ± 18.1	133.8 ± 24.8	.228	-8.90 (-24.48 – 6.67)					
Triglycerides (mg·dL-1)	PRED-G	100.0 (55.0, 131.0)	85.0 (69.0, 128.0)	.783	-1.00 # (-33.08 – 15.45)	.340	91.0 (51.0, 143.5)	79.0 (53.5, 128.0)	.109	-11.00 # (-28.86 – 4.55)
	HRV-G	90.5 (45.0, 187.8)	65.0 (41.0, 146.3)	.065	-14.50 # (-75.13 – 4.68)					
HDL-C (mg·dL-1)	PRED-G	39.7 ± 7.3	44.4 ± 8.0	.006*	4.64 (1.69 – 7.58)	.817	42.3 ± 10.8	47.3 ± 11.3	.008*	5.05 (1.47 – 8.63)
	HRV-G	45.0 ± 13.5	50.5 ± 13.8	.140	5.5 0 (-2.19 – 13.20)					
LDL-C (mg·dL-1)	PRED-G	67.5 ± 23.9	65.7 ± 25.7	.561	-1.73 (-8.13 – 4.68)	.419	70.7 ± 19.5	66.0 ± 20.2	.205	-4.71 (-12.22 – 2.79)
	HRV-G	74.3 ± 13.4	66.3 ± 13.3	.278	-8.00 (-23.67 – 7.67)					
Hemoglobin A1c (mmol·mol-1)	PRED-G	46.0 ± 6.7	40.5 ± 4.1	<.001*	-5.45 (-8.03 – -2.88)	.835	44.6 ± 6.2	39.3 ± 4.2	<.001*	-5.29 (-6.99 – -3.58)
	HRV-G	43.1 ± 5.7	38.0 ± 4.1	.002*	-5.10 (-7.85 – -2.35)					
Platelet	PRED-G	210.4 ± 46.2	204.1 ± 50.5	.292	-6.27 (-18.83 – 6.28)	.671	222.6 ± 48.9	214.0 ± 51.0	.128	-8.52 (-19.72 – 2.67)
	HRV-G	236.0 ± 50.6	225.0 ± 51.8	.286	-11.0 (-32.96 – 10.96)					
Red blood cells	PRED-G	5.0 ± 0.2	4.9 ± 0.3	.339	-0.06 (-0.19 – 0.07)	.524	4.9 ± 0.3	4.8 ± 0.3	.122	-0.10 (-0.22 – 0.03)
	HRV-G	4.8 ± 0.4	4.7 ± 0.3	.241	-0.14 (-0.39 – 0.11)					
Hemoglobin (g·dL-1)	PRED-G	15.2 (14.3, 15.7)	15.0 (14.1, 15.5)	.062	0.00 # (-0.60 – 0.19)	.542	14.7 (14.1, 15.6)	14.6 (13.9, 15.4)	.104	0.00 # (-0.25 – 0.25)
	HRV-G	14.4 (13.8, 15.2)	14.0 (13.7, 15.1)	.586	0.10 # (-0.84 – 0.77)					

CI, confidence interval; *CK*, creatine kinase; *HDL-C*, high-density lipoprotein cholesterol; *HRV-G*, heart rate variability-guided training group; *LDH*, lactate dehydrogenase; *LDL-C*, low-density lipoprotein cholesterol; *PRED-G*, predefined training group

Data at pre- and post-intervention are delivered as mean ± *SD* or median (25th and 75th percentiles); p^A and p^B values refer to within-group and between-group differences, respectively; * denotes $p \leq .050$; # denotes median change instead of mean change

Table S4 Effect of exercise-based cardiac rehabilitation on the quality of life

		Based on the training group (PRED-G, n = 11; HRV-G, n = 10)					All patients (n = 21)			
Variable	Group	Pre	Post	p^A	Change (95% CI)	p^B	Pre	Post	p^A	Change (95% CI)
Emotional score	PRED-G	5.9 (5.3, 6.6)	6.5 (5.5, 6.7)	.898	0.14 # (-0.02 – 0.75)	.173	6.2 (5.5, 6.6)	6.3 (5.6, 6.6)	.999	0.14 # (-0.09 – 0.35)
	HRV-G	6.3 (5.9, 6.6)	6.1 (5.8, 6.5)	.867	0.03 # (-0.56 – 0.31)					
Physical score	PRED-G	6.1 ± 0.6	6.5 ± 0.3	.085	0.32 (-0.05 – 0.70)	.398	6.1 ± 0.6	6.4 ± 0.4	.058	0.23 (-0.01 – 0.47)
	HRV-G	6.1 ± 0.7	6.2 ± 0.5	.432	0.13 (-0.22 – 0.47)					
Social score	PRED-G	6.4 ± 0.5	6.6 ± 0.3	.087	0.27 (-0.05 – 0.59)	.390	6.3 ± 0.6	6.5 ± 0.4	.025*	0.20 (0.03 – 0.37)
	HRV-G	6.3 ± 0.7	6.4 ± 0.5	.124	0.12 (-0.05 – 0.30)					
Global score	PRED-G	6.1 ± 0.7	6.4 ± 0.4	.067	0.33 (-0.03 – 0.69)	.187	6.1 ± 0.7	6.3 ± 0.5	.056	0.20 (-0.01 – 0.41)
	HRV-G	6.2 ± 0.7	6.2 ± 0.5	.565	0.06 (-0.17 – 0.30)					

CI, confidence interval; *HRV-G*, heart rate variability-guided training group; *PRED-G*, predefined training group

Data at pre- and post-intervention are delivered as mean ± *SD* or median (25th and 75th percentiles); p^A and p^B values refer to within-group and between-group differences, respectively; * denotes $p \leq .050$; # denotes median change instead of mean change

Table S5 Dietary intake

	PRED-G, n = 11		HRV-G, n = 10	
Variable	Pre	Post	Pre	Post
Energy (Kcal)	1325.8 (1245.1, 1406.4)	1278.3 (1116.8, 1576.7)	1264.9 (1134.6, 1466.3)	1221.5 (1185.4, 1489.1)
Water (mL)	2211.4 (1020.9, 2363.9)	2208.9 (1931.4, 2588.6)	1701.3 (996.7, 2443.3)	2270.0 (1812.2, 3239.3)
Protein (g)	83.4 (78.1, 90.0)	79.7 (69.6, 88.3)	81.4 (67.8, 89.3)	71.1 (62.8, 85.3)
Total fat (g)	41.9 (32.1, 54.8)	44.3 (34.3, 54.4)	47.3 (38.9, 54.0)	42.7 (34.8, 57.0)
Saturated (g)	10.7 (5.6, 13.4)	9.9 (8.0, 12.5)	10.3 (7.1, 12.9)	9.0 (7.5, 12.6)
Monounsaturated (g)	13.5 (9.4, 20.7)	15.2 (12.4, 18.2)	18.7 (11.9, 20.7)	17.4 (9.5, 21.0)
Polyunsaturated (g)	6.1 (5.2, 14.1)	7.4 (5.6, 15.8)	6.4 (4.8, 15.0)	6.1 (4.7, 16.4)
EPA (g)	0.28 ± 0.25	0.18 ± 0.22*	0.16 ± 0.12	0.18 ± 0.14
DHA (g)	0.49 (0.08, 0.86)	0.32 (0.08, 0.49)*	0.27 (0.21, 0.47)	0.31 (0.13, 0.49)
Cholesterol (mg)	283.1 ± 122.7	298.9 ± 101.2	297.7 ± 112.0	209.6 ± 74.9*
Carbohydrates (g)	155.2 ± 49.7	153.5 ± 40.5	158.9 ± 61.0	161.0 ± 37.2
Fiber (g)	19.6 ± 9.9	20.2 ± 11.5	21.0 ± 8.8	20.5 ± 8.6

DHA, docosahexaenoic acid; *EPA,* eicosapentaenoic acid; *HRV-G,* heart rate variability-guided training group; *PRED-G,* predefined training group

Data at pre- and post-intervention, as well as changes at follow-up are delivered as mean ± *SD* or median (25th and 75th percentiles); * refer to within-group and between-group differences, respectively

Supplemental references

1. Marfell-Jones, M. J.; Stewart, A. D.; De Ridder, J. H., International standards for anthropometric assessment. 2012.
2. Durnin, J. V.; Womersley, J., Body fat assessed from total body density and its estimation from skinfold thickness: measurements on 481 men and women aged from 16 to 72 years. Br J Nutr 1974, 32, (1), 77-97.
3. Martin, A., Anthropometric assessment of bone mineral. Anthropometric assessment of nutritional status. New York: Wiley-Liss 1991, 185-96.
4. Lee, R. C.; Wang, Z.; Heo, M.; Ross, R.; Janssen, I.; Heymsfield, S. B., Total-body skeletal muscle mass: development and cross-validation of anthropometric prediction models. Am J Clin Nutr 2000, 72, (3), 796-803.
5. Höfer, S.; Lim, L.; Guyatt, G.; Oldridge, N., The MacNew Heart Disease health-related quality of life instrument: a summary. Health Qual Life Outcomes 2004, 2, 3.
6. Chiavaroli, L.; Nishi, S. K.; Khan, T. A.; Braunstein, C. R.; Glenn, A. J.; Mejia, S. B.; Rahelić, D.; Kahleová, H.; Salas-Salvadó, J.; Jenkins, D. J. A.; Kendall, C. W. C.; Sievenpiper, J. L., Portfolio Dietary Pattern and Cardiovascular Disease: A Systematic Review and Meta-analysis of Controlled Trials. Prog Cardiovasc Dis 2018, 61, (1), 43-53.
7. Gargallo Fernández, M.; Basulto Marset, J.; Breton Lesmes, I.; Quiles Izquierdo, J.; Formiguera Sala, X.; Salas-Salvadó, J., Evidence-based nutritional recommendations for the prevention and treatment of overweight and obesity in adults (FESNAD-SEEDO consensus document). Methodology and executive summary (I/III). Nutr Hosp 2012, 27, (3), 789-99.
